# Autophagy Induced by HIF1α Overexpression Supports Trophoblast Invasion by Supplying Cellular Energy

**DOI:** 10.1371/journal.pone.0076605

**Published:** 2013-10-03

**Authors:** Mikiko Yamanaka-Tatematsu, Akitoshi Nakashima, Naonobu Fujita, Tomoko Shima, Tamotsu Yoshimori, Shigeru Saito

**Affiliations:** 1 Department of Obstetrics and Gynecology, Faculty of Medicine, University of Toyama, Toyama, Japan; 2 Department of Genetics, Osaka University Graduate School of Medicine, Suita, Osaka, Japan; University of Pittsburgh, United States of America

## Abstract

Extravillous trophoblasts (EVTs) characterize the invasion of the maternal decidua under low oxygen and poor nutrition at the early feto-maternal interface to establish a successful pregnancy. We previously reported that autophagy in EVTs was activated under 2% O_2_ *in vitro*, and autophagy activation was also observed in EVTs at the early feto-maternal interface *in vivo*. Here, we show that autophagy is an energy source for the invasion of EVTs. Cobalt chloride (CoCl_2_), which induces hypoxia inducible factor 1α (HIF1α) overexpression, activated autophagy in HTR8/SVneo cells, an EVT cell line. The number of invading HTR8-ATG4B^C74A^ cells, an autophagy-deficient EVT cell line, was markedly reduced by 81 percent with the CoCl_2_ treatment through the suppression of MMP9 level, although CoCl_2_ did not affect the cellular invasion of HTR8-mStrawberry cells, a control cell line. HTR8-ATG4B^C74A^ cells treated with CoCl_2_ showed a decrease in cellular adenosine triphosphate (ATP) levels and a compensatory increase in the expression of purinergic receptor P2X ligand-gated ion channel 7 (P2RX7), which is stimulated with ATP, whereas HTR8-mStrawberry cells maintained cellular ATP levels and did not affect P2RX7 expression. Furthermore, the decreased invasiveness of HTR8-ATG4B^C74A^ cells treated with CoCl_2_ was neutralized by ATP supplementation to the level of HTR8-ATG4B^C74A^ cells treated without CoCl_2_. These results suggest that autophagy plays a role in maintaining homeostasis by countervailing HIF1α-mediated cellular energy consumption in EVTs.

## Introduction

Trophoblast stem cells differentiate into two cell types in humans, villous trophoblasts and extravillous trophoblasts (EVTs). Villous trophoblasts are composed of cytotrophoblasts and syncytiotrophoblasts. During intact placentation, the placenta is constructed under low oxygen (2–5% O_2_) and low glucose concentrations (1 mM) until 11 weeks of gestation [1], [2], [3], [4]. Intra-placental oxygen tension is rapidly increased after 12 weeks because endovascular EVTs invade the uterine spiral arteries, replace endothelial cells, and participate in the degradation of tunica media smooth muscle cells [5]. This remodeling of the spiral arteries is essential for allowing proper placental perfusion to sustain fetal growth. Failed interstitial and endovascular trophoblast invasion may lead to inadequate transformation of the spiral arteries, resulting in preeclampsia or fetal growth restriction (FGR) [6], [7], [8]. However, the causes of invasion failure by EVTs in early human placenta are still unknown.

Autophagy is an intracellular bulk degradation system through which cytoplasmic components are degraded in lysosomes, resulting in energy production [9]. It occurs ubiquitously at basal levels in all eukaryotic cells and contributes to the routine turnover of cytoplasmic components. The autophagy activation in response to environmental stress is required for cell survival in yeast as well as in mammals [10]. Hypoxia inducible factor (HIF) is well known to be a central transcription factor that enables adaptive responses to hypoxic stress under normal and pathological conditions by activating a large number of genes responsible for oxygen delivery, angiogenesis, cell proliferation, cell differentiation, and metabolism [11], [12]. Phenotypic changes in EVTs, from a proliferative type to an invasive type, in the early placenta are well known to be regulated through HIF1 transcription factors, which suggests that HIF1α is a master regulator for EVT differentiation [13]. Recent reports have indicated that hypoxia can activate a lysosomal degradation pathway known as autophagy, which mediates both selective and bulk degradation of proteins, cytoplasmic content, and organelles. Regarding the correlation between autophagy and EVT functions, we previously reported that autophagy was essential for the invasion of EVTs and EVT-vascular remodeling under physiological hypoxia, which is necessary for the first stage of placentation *in vitro* and *in vivo* [14]. Autophagy also plays an increasingly recognized role in quality control during hypoxia by removing mitochondria that may otherwise become cytotoxic [15]. The HIF1α expression levels in the human placenta are high at 7–9 weeks of gestation when oxygen tension is low, and decrease at around 12 weeks of gestation when placental oxygen tension increases [16]. However, continuous exposure to hypoxia in the early stage of pregnancy has been shown to induce preeclampsia-like symptoms in IL-10 knockout mice [17], suggesting that severe hypoxia itself could cause preeclampsia. During early-onset human preeclampsia, the placenta is exposed to severe hypoxia independently of intervillous maternal blood-oxygen tension, due to a loss of the placenta's ability to adapt to variations in oxygen tension [18]. Although we have reported that impairment of autophagy by soluble endoglin contributes to invasion failure under physiological hypoxia, it remains unclear how severe hypoxia, which is lower than physiological hypoxia, affects the functions in EVTs with or without autophagy.

In this study we show that overexpression of HIF1α decreases the invasiveness of autophagy-deficient HTR8/SVneo cells by suppressing cellular adenosine triphosphate (ATP) levels. Autophagy-deficient HTR8/SVneo cells with overexpression of HIF1α also expressed purinergic receptor P2X ligand-gated ion channel 7 (P2RX7). Furthermore, ATP treatment recovered the invasive nature of autophagy-deficient HTR8/SVneo cells. These results suggest that autophagy supplies cellular energy for EVTs to protect them from HIF1α-induced energy depletion.

## Materials and Methods

### Reagents and antibodies

CoCl_2_ (Fluka Biochemika Ltd., Buchs, Switzerland) was purchased from Fluka Biochemika Ltd.. Rpamycin (R8781, 100 or 500 nM), an activator of autophagy, and three-methyladenine (3-MA, 5 mM, M9281), an inhibitor of autophagy, were purchased from Sigma-Aldrich (St. Louis, MO, USA). The following antibodies (Ab) were used: rabbit polyclonal Ab for MAP1LC3B (PM036, MBL, Nagoya, Japan), rabbit monoclonal Ab for P2RX7 (ab109246, Abcam Inc., Cambridge, MA, USA), mouse monoclonal Ab for HIF1-α (H72320, BD Pharmingen, Franklin Lakes, NJ, USA) and mouse monoclonal Ab for α-tubulin (T8203, Sigma-Aldrich). The protease inhibitors E64d (4321-v Peptide Institute, Osaka, Japan) and pepstatin A (4397, Peptide Institute) were purchased from the Peptide Institute Inc.

### Cell culture

The EVT cell lines HTR8/SVneo (a gift from Dr. Charles H. Graham, Department of Anatomy and Cell Biology, Queen's University, Ontario, Canada) and HchEpC1b were used in this study [19], [20]. The constructed autophagy-deficient cell line, HTR8-ATG4B^C74A^ mutant cells, and the control vector-infected cell line, HTR8-mStrawberry cells, were also used. The procedures for constructing the vectors were reported previously [21]. The expression of mStrawberry was confirmed by fluorescence microscopy. HTR8/SVneo cells were cultured in DMEM supplemented with 10% fetal bovine serum (FBS), 100 U/ml penicillin and 100 µg/ml streptomycin (15140, Life Technologies, Carlsbad, CA, USA) at 37°C in a 5% CO_2_ atmosphere. HchEpC1b cells were cultured in RPMI1640 supplemented with 10% FBS, 100 U/ml penicillin and 100 µg/ml streptomycin. To mimic severe hypoxic conditions, cells were plated on a 35-mm dish at 2×10^5^ cells/dish, and, after 24 h, were cultured in medium containing CoCl_2_ (250 µM, Fluka Biochemika Ltd.) under a 5% CO_2_ atmosphere at 37°C.

### Quantitative analysis of GFP-LC3 puncta

For the quantitative analysis of MAP1LC3B (LC3), the cells were pretreated with the lysosomal protease inhibitors E64d (10 ng/ml) and pepstatin A (10 ng/ml) for 2 h to distinguish cytoplasmic LC3 puncta, and were then fixed with 4% paraformaldehyde-PBS [22]. Cells were subsequently stained with the LC3 antibody. The incidence of autophagy was estimated by quantifying the number of LC3 puncta within LC3-stained cells by manually counting five independent visual fields using a confocal microscope (LSM700, Carl Zeiss, Oberkochen, Germany). At least 5 cells per 40 high power fields were counted in ten randomly chosen fields, and these experiments were independently performed at least three times.

### Invasion assay

**Figure 1:**
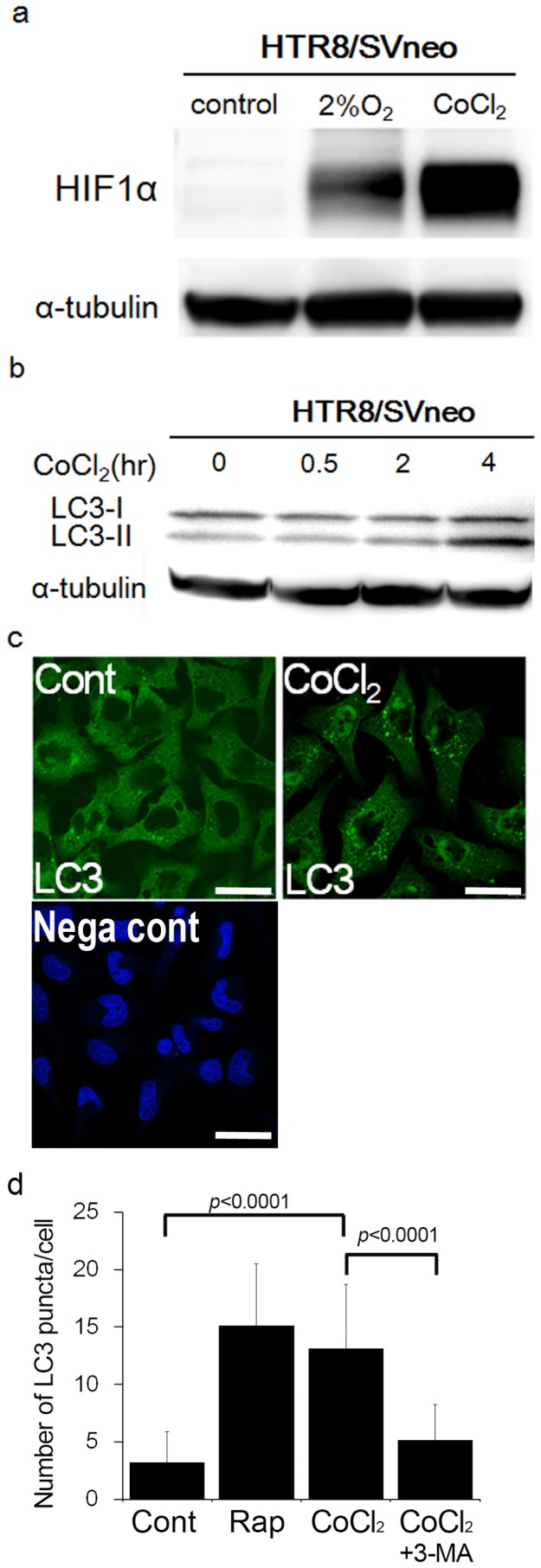
Estimation of autophagy in HTR8/SVneo cells, when CoCl_2_ induced HIF1α expression. a) Western blots in HTR8/SVneo cells under 250 µM CoCl_2_, 2% oxygen tension, or DMSO (control) for 24 h were shown as follows: HIF1α and α-tubulin. b) The expression of MAP1LC3B-I and -II (LC3-I and LC3-II) in HTR8/SVneo cells was examined in the presence of E64d and pepstatin under 250 µM CoCl_2_ for the indicated times. The expression of α-tubulin was used as an internal control. c) Representative panels show anti-LC3 staining in HTR8/SVneo cells under DMSO (control) or 250 µM CoCl_2_ for 24 h. Negative control (Nega cont) was treated with rabbit serum instead of anti-LC3 antibody and stained with DAPI (4′, 6-diamidino-2-phenylindole). Scale bar: 30 µm. d) The graph indicates the number of LC3 puncta in HTR8/SVneo cells under DMSO (control), rapamycin (500 nM), 250 µM CoCl_2_, or 250 µM CoCl_2_ with 5 mM 3-MA for 24 h. These experiments were independently performed at least three times.

**Figure 2:**
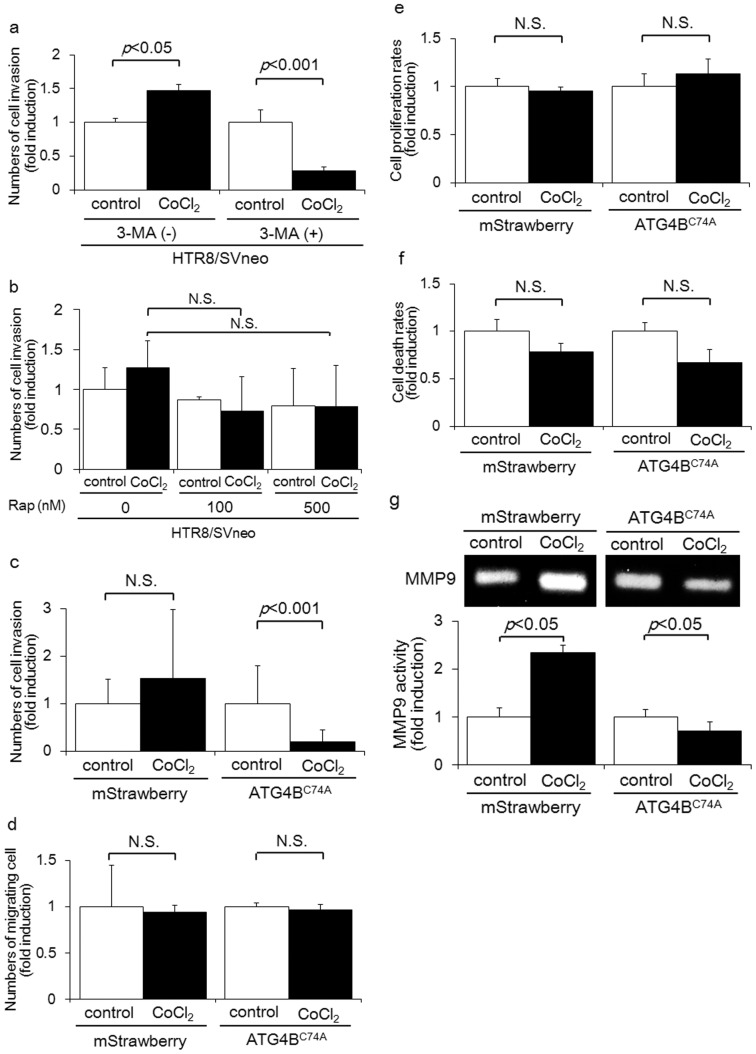
Impairment of autophagy resulted in EVT invasion failure with the CoCl_2_ treatment via the suppression of MMP9. a) Invasion assays were performed with HTR8/SVneo cells in the presence or absence of three methyladenine (3-MA, 5 mM) under DMSO (control: white bars) or 250 µM CoCl_2_ (black bars) for 24 h. The *Y*-axis indicates the number of invading cells. b) Invasion assays were performed with HTR8/SVneo cells in the presence of 100 nM or 500 nM rapamycin under DMSO (control: white bars) or 250 µM CoCl_2_ (black bars) for 48 h. c) Invasion assays were performed with HTR8-Atg4B^C74A^ mutant cells, an autophagy-deficient EVT cell line, or HTR8-mStrawberry cells, the control cell line, under DMSO (control: white bars) or 250 µM CoCl_2_ (black bars) for 48 h. The *Y*-axis indicates the number of invading cells. d) Migration assays were performed with HTR8-Atg4B^C74A^ mutant cells or HTR8-mStrawberry cells under DMSO (control: white bars) or 250 µM CoCl_2_ (black bars) for 48 h. The *Y*-axis indicates the number of migrating cells. e) Cell proliferation rates were estimated by the WST-1 assay in HTR8-Atg4B^C74A^ mutant cells or HTR8-mStrawberry cells under DMSO (control: white bars) or 250 µM CoCl_2_ (black bars) for 48 h. f) Cell death rates were estimated by 5 µg/ml propidium iodide (PI) staining in HTR8-Atg4B^C74A^ mutant cells or HTR8-mStrawberry cells under DMSO (control: white bars) or 250 µM CoCl_2_ (black bars) for 48 h. g) Representative gelatin zymograms showed MMP9 secreted from HTR8-Atg4B^C74A^ mutant cells or HTR8-mStrawberry cells after 48 h culture in DMSO or 250 µM CoCl_2_. For all samples, 20 mg total protein was loaded per well. The bars indicate the median values of MMP9 in each group. Data were normalized to the protein concentrations of the culture media for each sample. (c-g) Fold inductions were calculated in comparison to the number of HTR8 cells treated with DMSO as one. Data were shown as the mean ± S.E. of three independent experiments. N.S.: not significant

An invasion assay was performed using a BD BioCoat Growth Factor Reduced Matrigel Invasion Chamber (354483, BD Biosciences, San Jose, CA, USA) according to the manufacturer's instructions. Cells were plated in the upper insert at 5×10^4^/well and incubated in DMEM with or without CoCl_2_ (250 µM). When HTR8/SVneo cells were treated with CoCl_2_, the expression of HIF-1α was increased in a dose-dependent manner (Figure S1a). To analyze the effect of severe hypoxia in HTR8/SVneo cells, 250 µM CoCl_2_, which has been shown to induce higher HIF-1α expression than under a 2% oxygen tension condition (Fig. 1a) without affecting proliferation (Fig. 2e), was used in our assay. These cells were incubated for 24 or 48 h, and then the upper surface of the membrane in each insert was gently scrubbed with a cotton swab to remove all of the non-invading cells. Cells on the under surfaces of the membranes were fixed in 100% methanol (131-01826, Wako Pure Chemical Industries Ltd., Osaka, Japan), and stained with 0.05% Toluidine Blue Solution (206-14555, Wako Pure Chemical Industries Ltd.). For each experiment, the number of cells in seven randomly chosen fields of each filter was estimated by manual counting, and these experiments were independently performed at least three times.

### Transwell migration assay

Cell migration assay was carried out using the cell migration assay system (CBA-100, Cell Biolabs, Inc., San Diego, CA, USA) equipped with 8-µm pore size of migration chambers. Methods used in this assay were similar to Matrigel invasion assay except that the transwell insert was not coated with Matrigel. After 48 h of incubation, migrated cell numbers were calculated. For each experiment, the number of cells in seven randomly chosen fields of each filter was estimated by manual counting, and these experiments were independently performed at least three times.

### Western blotting

Cells were washed with cold PBS, harvested and lysed in a lysis buffer (25 mM HEPES-NaOH, 1 mM EDTA, 1 mM DTT, 400 mM NaCl, 1.5 mM MgCl_2_, 0.1% Triton X-100, 0.1% protease inhibitor cocktail (Sigma-Aldrich), and 0.1% PMSF (Sigma-Aldrich)). The lysates were centrifuged at 14000 rpm for 15 min at 4°C, and the protein concentration of the supernatant was determined with a BCA protein assay kit (Bio-Rad Laboratories Inc., Hercules, CA, USA) according to the manufacturer's instructions. Protein samples were mixed with a 2×sample buffer (Wako Pure Chemical Industries Ltd.), and samples were heated at 95°C for 3 min. Equal amounts of protein were then subjected to 15% sodium dodecyl sulfate-polyacrylamide gel electrophoresis (SDS-PAGE) and transferred to polyvinylidene difluoride membranes. The membranes were then incubated overnight at 4°C in a blocking buffer (tris buffered saline with 0.1% Tween 20 and 5% bovine serum albumin; 5% BSA/TBST), before being washed with PBST and then incubated for 2 h at room temperature with the following antibodies: anti-LC3 (1∶1000; MBL), anti-HIF1α (1∶500; BD Pharmingen), and anti-α-tubulin (1∶1000; Sigma-Aldrich, diluted with 5% BSA/TBST). Finally, they were incubated for 1 h with secondary antibodies labeled with horseradish peroxidase-conjugated anti-mouse or anti-rabbit antibody (1∶1000; Cell Signaling, Beverly, MA, USA) and visualized with an enhanced chemiluminescence detection system (ECL detection kit; PIERCE, Rockford, IL, USA).

### Flow cytometry

Cells were harvested, washed once, and fixed in 4% paraformaldehyde-PBS for 15 min at room temperature. Cells were then resuspended in PBS solution containing the anti- P2RX7 antibody (1∶100; Abcam Inc.) for 30 min at room temperature. Cells were subsequently stained with FITC goat anti-rabbit IgG (1∶50; BD Pharmingen) and incubated for 30 min at room temperature. Samples were then analyzed with a FACS Calibur flow cytometer (BD Biosciences) using Cell Quest software as described previously (BD Biosciences) [23]. Negative controls were produced by replacing the primary antibody with normal rabbit serum. To estimate the proportion of dead cells, the harvested cells were suspended in PBS solution containing 5 µg/ml propidium iodide (P3566, Molecular Probes, Eugene, OR, USA), and were incubated for 30 min at room temperature. Samples were also analyzed with a FACS Calibur flow cytometer.

### Cell proliferation assay

A cell proliferation assay was performed with the cell proliferation reagent WST-1 (Roche, Basel, Switzerland) according to the manufacturer's instructions. 2×10^4^ cells/well were incubated in microplates for 24 or 48 h (tissue culture grade, 96 wells, flat bottom Falcon) at a final volume of 100 µl/well for each culture medium. After the addition of 10 µg/well of the WST-1 reagent to each well, these cells were further incubated for and shaken thoroughly for 1 min on a shaker. The appropriate cell number was determined based on the several control concentrations of cells used in the experiment. We then measured the absorbance of the samples at 450 nm (reference wavelength 690 nm) against the background control, using a multiwall plate reader.

### Gelatin zymography

To determine the levels of secreted proteases, zymographic analysis was performed using a gelatin-zymography kit (Primary Cell Co. Ltd., Hokkaido, Japan) according to the manufacturer's instructions. Conditioned medium samples were collected, and 20 mg total protein (determined by the BioRad Protein Assay; BioRad Laboratories) was resolved in the sample buffer. Equal amounts of protein were applied and then electrophoresed for 180 min. The gels were then washed in 2.5% Triton X-100 to remove the SDS and incubated overnight at 37°C in a solution of 50 mM Tris and 5 mM CaCl_2_ to allow the enzymes to digest the substrate. After incubation, the gels were stained with Coomassie Brilliant Blue R250, then destained, preserved, and dried. Dried gels were then scanned, and densitometry was performed. In this assay, the predominant form of MMP9 was the latent form, and the MMP9 level was assessed by densitometry.

### ATP assay

The ATP Bioluminescence Assay Kit HS II (Roche) was used for the ATP assay according to the instruction manual. To briefly summarize, cells were treated for 48 h, harvested at 4°C, and resuspended at 1,000 cells/µl in lysis buffer. Cells were then boiled for 10 min, cooled on ice for 30 s, and spun at 10,000 Xg for 5 min at 4°C. The cleared supernatant was then used to determine ATP concentrations, and the measured ATP concentrations of the supernatants were normalized to their protein concentrations. This experiment was repeated at least three times.

### Statistical Analysis

Results are presented as the mean ± S. E. and comparisons between multiple groups were evaluated with two-way ANOVA. When a significant difference was detected, a post-hoc test was further performed. Values of *p*<0.05 were considered significant.

## Results

### Overexpression of HIF1α by cobalt chloride (CoCl_2_) activated autophagy in HTR8/SVneo cells

We previously reported that hypoxia induces hypoxia inducible factor 1α (HIF1α), and activates autophagy in EVT cell lines and EVT primary cells [14]. However, it remains to be seen whether the overexpression of HIF1α activates autophagy or affects EVT functions. To verify the effects of overexpressed HIF1α on EVT functions, HTR8/SVneo cells, an EVT cell line, were treated with cobalt chloride (CoCl_2_). This stabilized HIF1α and induced HIF1 responsive genes with kinetics similar to those of hypoxia [24]. HTR8/SVneo cells were treated with 250 µM of CoCl_2_, which induced a higher expression of HIF1α than that produced by a 2% oxygen concentration condition (a physiological oxygen concentration in the placenta during early pregnancy, Fig. 1a). Next, we examined whether the overexpression of HIF1α with CoCl_2_ induced autophagy in HTR8/SVneo cells. The conversion of LC3-I into LC3-II, a marker for autophagy activation, was examined by western blotting [21]. As depicted in Figure 1b, the amount of LC3-II increased with the 4 h of CoCl_2_ treatment. Furthermore, we monitored the distribution of the LC3 protein and observed LC3-II punctate formation, which indicates the presence of autophagosome (Fig. 1c). The number of LC3 puncta was significantly higher in HTR8/SVneo cells treated with CoCl_2_ for 24 h compared to the control, as well as in the rapamycin treatment, which induces autophagy by inhibiting the mammalian target of rapamycin (mTOR) (Fig. 1d, *p*<0.0001). On the other hand, CoCl_2_-mediated LC3 punctation was significantly suppressed by three-methyladenine (3-MA), an inhibitor of autophagy, in HTR8/SVneo cells (Fig. 1d, *p*<0.0001). Collectively, these results suggested that CoCl_2_, which induced higher expression of HIF1α, also induced autophagy in HTR8/SVneo cells.

### Overexpression of HIF1α decreased cell invasion in autophagy-deficient HTR8/SVneo cells

To explore the role of autophagy in EVT cell invasion, invasion assays were performed with 3-MA. 3-MA clearly suppressed the number of invading HTR8/SVneo cells in the presence, but not absence, of CoCl_2_ after 24 h (Fig. 2a). On the other hand, rapamycin did not accelerate the number of invading HTR8/SVneo cells in the presence or absence of CoCl_2_ after 48 h (Fig. 2b). These results suggest that autophagy inhibition, but not autophagy activation, is critical for the invasion of EVTs with CoCl_2_ treatment. In order to clarify the role of autophagy inhibition in EVT invasion when HIF1α was overexpressed in EVTs, invasion assays were performed in HTR8-ATG4B^C74A^ mutant cells, an autophagy-deficient EVT cell line, and HTR8-mStrawberry cells, a control cell line. Before these procedures, there were no significant differences in the level of HIF1α expression induced by CoCl_2_ between HTR8-ATG4B^C74A^ mutant cells and HTR8-mStrawberry cells (Fig. 1a and Fig. S1b). The number of invading HTR8-ATG4B^C74A^ mutant cells was markedly reduced by 81 percent with the CoCl_2_ treatment after 48 h (p<0.001); on the other hand, the number of invading HTR8-mStrawberry cells did not change (Fig. 2c). Interestingly, the number of migrating HTR8-ATG4B^C74A^ mutant and HTR8-mStrawberry cells remained stable in the presence and absence of the CoCl_2_ treatment after 48 h (Fig. 2d). Consequently, we investigated how autophagy deficiency affected cell growth and cell death, in conjunction with the CoCl_2_ treatment. No significant difference was observed in the cell growth rate between the HTR8-ATG4B^C74A^ mutant cells cultured with or without CoCl_2_ for 48 h (Fig. 2e). Similar results were obtained for HTR8-mStrawberry cells. The CoCl_2_ treatment had no effect on the cell death rate of HTR8-ATG4B^C74A^ mutant or HTR8-mStrawberry cells (Fig. 2f). To investigate the mechanism by which autophagy affects trophoblast invasion in the presence of CoCl_2_, gelatin zymography was performed to estimate the expression of matrix metalloproteinases (MMP). The expression of MMP9 in HTR8-mStrawberry cells treated with CoCl_2_ was significantly higher than those not subjected to this treatment (Fig. 2g, *p*<0.05). On the other hand, MMP9 levels in HTR8-ATG4B^C74A^ mutant cells were significantly lower in cells with the CoCl_2_ treatment than in those without (Fig. 2g, *p*<0.05). Expression levels of MMP2 in the culture media obtained from HTR8-ATG4B^C74A^ mutant or HTR8-mStrawberry cells were low. These results suggest that autophagy deficiency markedly reduces the invasion of EVTs, at least in part by inhibiting the expression of MMP 9 when HIF1α is overexpressed in EVTs.

### Attenuation of cellular ATP levels and increased P2RX7 expression in autophagy-deficient HTR8/SVneo cells

**Figure 3:**
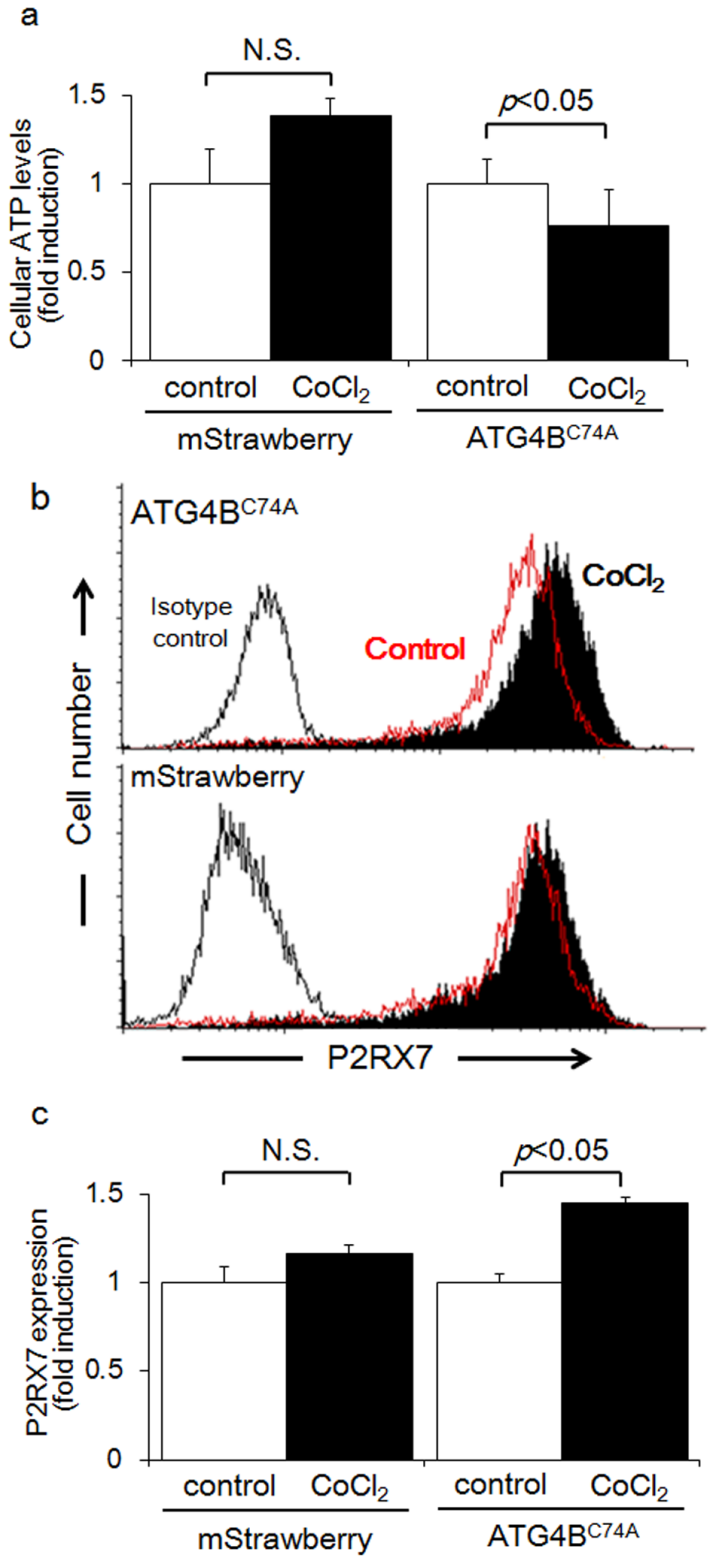
CoCl_2_ decreased cellular ATP levels, but increased P2RX7 expression in autophagy-deficient HTR8/SVneo cells. a) Cellular ATP levels in HTR8-Atg4B^C74A^ mutant cells, the autophagy-deficient EVT cell line, or HTR8-mStrawberry cells, the control cell line, were determined after the treatment with DMSO (control: white bars) or 250 µM CoCl_2_ (black bars) for 48 h. b) P2RX7 expression in HTR8-Atg4B^C74A^ mutant cells (upper panel) or HTR8-mStrawberry cells (lower panel) treated with DMSO (red lines) or 250 µM CoCl_2_ (black solids) were determined by flow cytometry for 48 h. Isotype controls are shown as black lines. c) P2RX7 expression was estimated by the mean fluorescent intensity in HTR8-Atg4B^C74A^ mutant cells or HTR8-mStrawberry cells treated with DMSO (control: white bars) or 250 µM CoCl_2_ (black bars) for 48 h. These experiments were independently performed at least three times. N.S.: not significant

Autophagy was originally discovered to be part of the machinery for producing energy in cells under conditions of poor nutrition. Cellular ATP levels have also been used as an indicator of cellular energy levels. The cellular ATP levels of HTR8-mStrawberry cells, the control cell line, cultured with CoCl_2_ did not change, compared against those cultured without CoCl_2_ for 48 h (Fig. 3a, left). On the other hand, cellular ATP levels were 0.76-fold lower in HTR8-ATG4B^C74A^ mutant cells, the autophagy-deficient EVT cell line, cultured with CoCl_2_ than in those without CoCl_2_ at 48 h (Fig. 3a, right, p<0.05). In association with ATP levels, we estimated the expression of purinergic receptor P2X ligand-gated ion channel 7 (P2RX7) is stimulated with ATP [25]. CoCl_2_ significantly augmented P2RX7 expression in HTR8-ATG4B^C74A^ mutant cells, whereas P2RX7 expression levels in HTR8-mStrawberry cells were unchanged (Fig. 3b). The mean fluorescent intensity of P2RX7 was significantly increased with CoCl_2_ treatment in HTR8-ATG4B^C74A^ mutant cells, but not in HTR8-mStrawberry cells (Fig 3c, p<0.05). Similar results were obtained in the other EVT cell line, HchEpC1b (Figure S2). These results indicate that overexpressed HIF-1α lead to decrease in cellular ATP levels and an increase in P2RX7 expression in HTR8-ATG4B^C74A^ mutant cells, but not in HTR8-mStrawberry cells.

### ATP recovered the suppression of HTR8-ATG4B^C74A^ mutant cell invasion with the CoCl_2_ treatment

**Figure 4:**
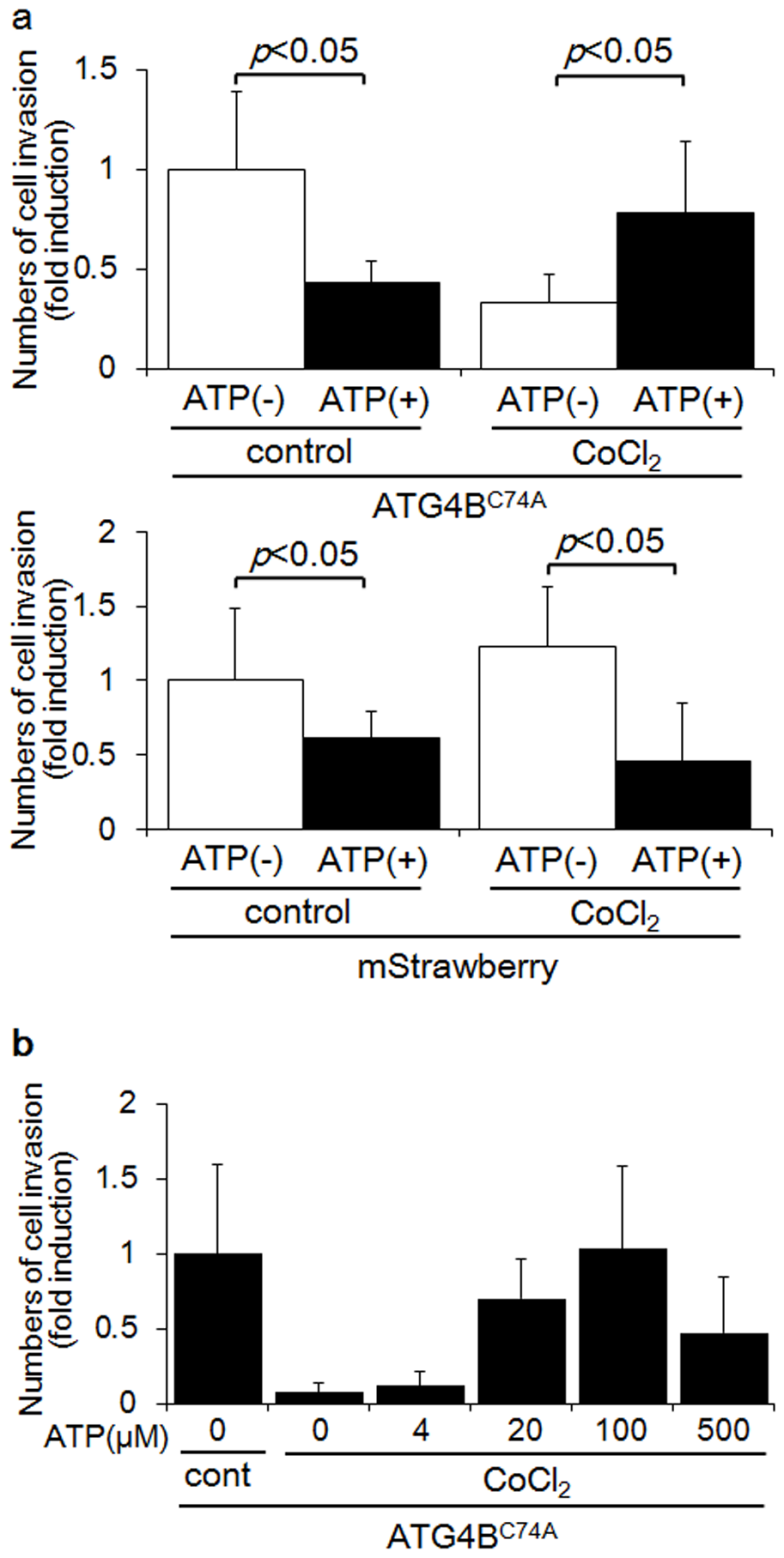
ATP supplementation recovered the suppression of invasion in autophagy-deficient HTR8/SVneo cells. a) Invasion assays were performed with HTR8-Atg4B^C74A^ mutant cells (upper panel), the autophagy-deficient EVT cell line, or HTR8-mStrawberry cells (lower panel), the control cell line, under DMSO (control) or 250 µM CoCl_2_ in the presence (black bars) or absence (white bars) of 100 µM ATP for 48 h. The *Y*-axis indicates the number of invading cells. b) Invasion assays were performed with HTR8-Atg4B^C74A^ mutant cells under 250 µM CoCl_2_ in the presence of increasing concentrations of ATP, as indicated for 48 h. The *Y*-axis indicates the number of invading cells. These experiments were independently performed at least three times.

The invasion assay was performed to clarify whether ATP supplementation rescued cell invasion. In HTR8-ATG4B^C74A^ mutant cells treated with CoCl_2_, the number of invading cells was significantly increased by ATP supplementation (Fig. 4a, upper right, *p*<0.05). On the other hand, ATP supplementation inhibited trophoblast invasion in HTR8-ATG4B^C74A^ mutant cells without CoCl_2_ treatment (Fig. 4a, upper left, *p*<0.05). ATP supplementation also inhibited trophoblast invasion in HTR8-mStrawberry cells, the control cell line, with or without CoCl_2_ treatment (Fig. 4a, lower panels, *p*<0.05). Thus, ATP supplementation recovered trophoblast invasion only in HTR8-ATG4B^C74A^ mutant cells treated with CoCl_2_. Invasion assays were performed in culture media supplemented with several concentrations of ATP to further clarify the role of ATP in trophoblast invasion. The number of invading cells was significantly increased in an ATP-dose dependent manner in Atg4B^C74A^ mutant cells with CoCl_2_ (Fig. 4b). Supplementation with 100 µM of ATP recovered the invasion of EVTs to the levels seen without CoCl_2_ treatment in HTR8-ATG4B^C74A^ mutant cells. However, ATP-recovered EVT invasion was attenuated at 500 µM ATP. Collectively, the overexpression of HIF1α decreased cellular energy in EVTs with an impaired autophagy status, while ATP supplementation recovered the invasion of EVTs by refilling energy levels.

## Discussion

We previously reported that autophagy was essential for the invasion of EVTs and EVT-vascular remodeling under a 2% oxygen concentration *in vitro* [14]. In this study, the invasiveness of autophagy-deficient HTR8/SVneo cells was markedly attenuated by overexpression of HIF1α through the suppression of MMP 9. Secondly, autophagy supplied cellular energy to assist HTR8/SVneo cell invasion when HIF1α overexpression was induced in HTR8/SVneo cells through treatment with CoCl_2_. Autophagy-deficient HTR8/SVneo cells overexpresseing HIF1α also showed increased P2RX7 expression. Finally, it was found that ATP recovered the invasiveness of autophagy-deficient HTR8/SVneo cells by refilling energy levels.

Chen et al recently reported that autophagy plays distinct roles for cell survival in endothelial cells between short term exposure and prolonged exposure to hypoxia. This reveals that hypoxia-induced autophagy enhances cell survival in the early hypoxic stages, but attenuates cell death in late hypoxic stages [26]. Regarding the EVT cell invasion, our previous report showed that the number of invading HTR8-ATG4B^C74A^ mutant cells, an autophagy-deficient EVT cell line, showed no difference between 2% O_2_ and 20% O_2_. The number of invading HTR8-mStrawberry cells, our control EVT cell line, was significantly higher in the short term when subjected to a 2% O_2_ condition than under 20% O_2_. In this study, the number of invading HTR8-mStrawberry cells did not increase when CoCl_2_ was used to induce HIF1α overexpression in HTR8-mStrawberry cells, and the number of invading HTR8-ATG4B^C74A^ mutant cells significantly decreased in the presence of CoCl_2_ (when exposed to treatment for 48 h under prolonged hypoxia) (Fig. 2c). The drastic decrease in the invasion of HTR8-ATG4B^C74A^ mutant cells at 48 h might rely on an absence of autophagy under conditions of prolonged hypoxia. On the other hand, it is possible that autophagy might protect the invasion of HTR8-mStrawberry cells against prolonged hypoxia. Thus, hypoxia-mediated autophagy may accelerate EVT invasion in the early stages of hypoxia conditions, and serve to maintain invasive cell character in the late stages of hypoxia. In addition, the increase of MMP9 expression that was observed in HTR8-mStrawberry at 48 h (Fig. 2g), might be reflected by a temporal increase of MMP9 after 24 h, but no significant difference between control and CoCl_2_ treatment conditions in HTR8-mStrawberry was seen at 72 h (data not shown).

Choi et al showed that a decrease in HIF1α expression mediated by siRNA markedly reduced the invasiveness of HTR8/SVneo cells after 24 h [27]. Our results showed that overexpression of HIF1α for 48 h inhibited the invasion of EVTs. These results suggest that the level of HIF1α expression coordinates EVT cell invasion. Furthermore, the number of invading HTR8-mStrawberry cells treated with 3-MA, an autophagy inhibitor, was markedly decreased in the presence of CoCl_2_, but not in the absence of CoCl_2_. Macroautophagy works constitutively to maintain homeostasis, meanwhile autophagy is further activated in response to severe stress, such as severe hypoxia or oxidative stress. We thought that activated autophagy contributed to the enhanced invasion of HTR8/SVneo cells under hypoxia, but basal autophagy did not affect the invasion of HTR8/SVneo cells under normoxia. Our results might suggest the importance of hypoxia-induced autophagy for the invasion of HTR8/SVneo cells, because large quantities of autophagy are induced under a hypoxic condition, but only small quantities occur under normoxia. On the other hand, rapamycin, an inducer of autophagy, did not increase the number of invading HTR8-mStrawberry cells, even in the absence of CoCl_2_. This suggests that phosphatidylinositol-3 kinase, but not mTOR, plays a critical role in the invasion of EVTs.

A previous study showed that the cell-permeable form of pyruvate, methylpyruvate, restored cellular ATP levels in cells with autophagy induced by growth factor deficiency [28]. Regarding the invasion of EVTs, the suppression of cellular ATP levels was correlated with the inhibition of invasion when HIF1α was overexpressed in HTR8-ATG4B^C74A^ mutant cells. Only ATP supplementation, not supplementation with methylpyruvate (MP), recovered HTR8-ATG4B^C74A^ mutant cell invasion characteristics when these cells were treated with CoCl_2_ (Figure S3). This occurred because hypoxia blocks ATP production in the tricarboxylic acid cycle and causes the cell to switch to the glycolytic cycle for cellular energy production. Though these results showed that autophagy activation may contribute to obtaining cellular energy during the invasion of EVTs, it remains unknown how ATP supplementation affected the invasion characteristic of EVTs. The number of invading HTR8-ATG4B^C74A^ mutant cells was recovered to control levels with 100 µM ATP supplementation. On the other hand, 100 µM ATP showed cytotoxicity for the invading HTR8-mStrawberry cells. A recent study showed that different concentrations of extracellular ATP served to modulate CD4^+^ T cells, according to their activated/regulatory status [29]. Interestingly, 250 nM ATP stimulated proliferation, cytokine release and the expression of adhesion molecules, while 1 mM ATP induced apoptosis and inhibited activated CD4^+^ T cell function. In contrast, 1 mM ATP enhanced the proliferation, adhesion, migration, and immunosuppressive ability of regulatory T cells, suggesting that reactivity to ATP is dependent on cell type. Additionally, the up-regulation of P2RX7 was detected in HTR8-ATG4B^C74A^ mutant cells, but not in HTR8-mStrawberry cells. Since P2RX7 mediates cellular energy by ingesting NADH [30], [31], it is assumed that the up-regulation of P2RX7 expression compensated for the down-regulation of cellular ATP levels in EVTs. As 500 µM ATP was more cytotoxic for the invading HTR8-ATG4B^C74A^ mutant cells than a dose of 100 µM ATP, it serves to reason that an appropriate concentration of ATP may be useful for recovering the invasion of EVTs.

**Figure 5:**
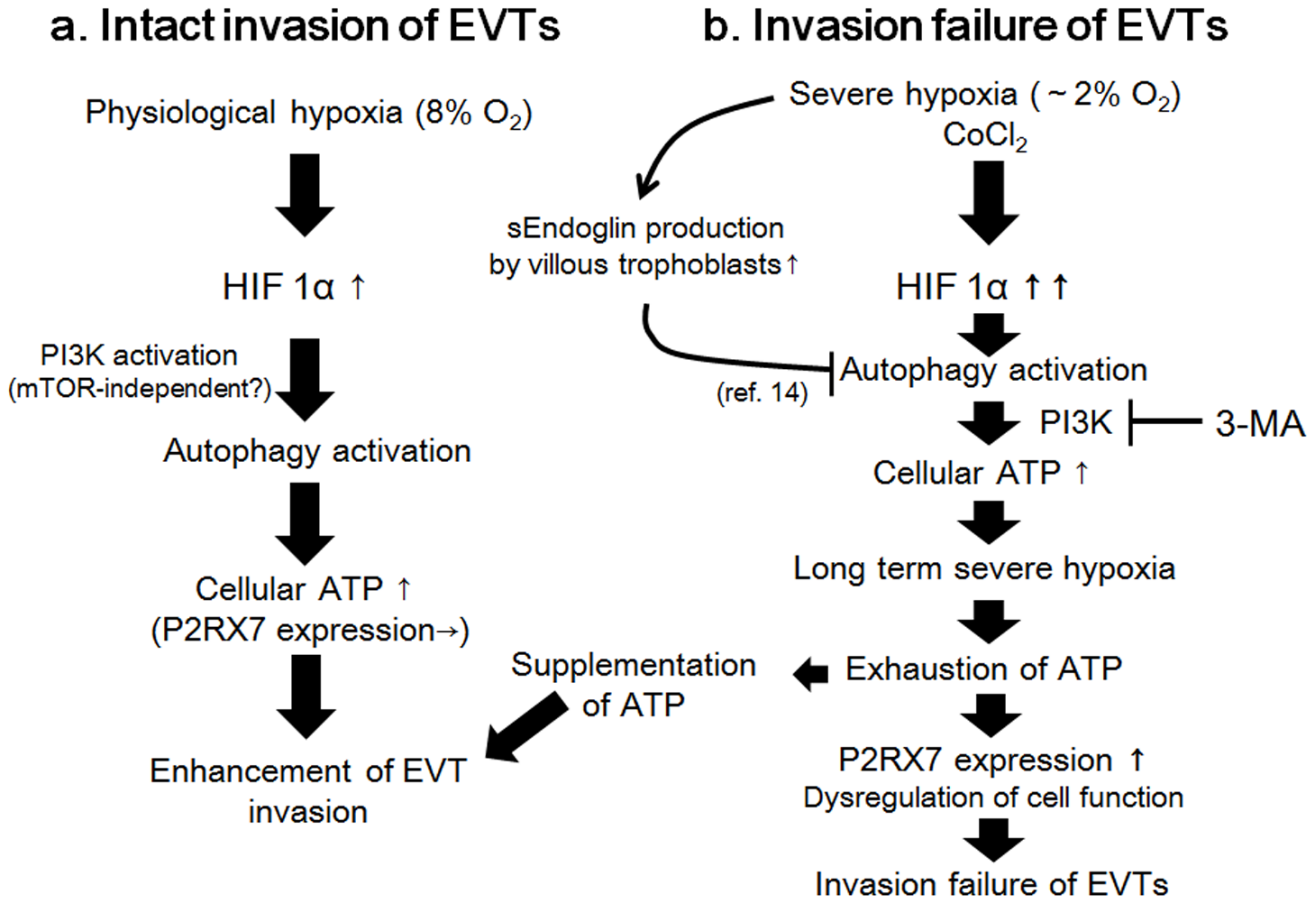
Correlation chart between autophagy and HIF1α for the invasion of EVTs. (a) In an intact invasion of EVTs, HIF1α activates autophagy via the PI3K pathway. Autophagy then supplies cellular energy to enhance the invasion of EVTs. (b) In an invasion failure, severe hypoxia or long term hypoxia may accelerate HIF1α overexpression in EVTs. EVTs with an impaired autophagy status by soluble endoglin did not produce energy for the invasion of EVTs with HIF1α overexpression, resulting in the inhibition of EVT invasion. Compensatory P2RX7 expression was enhanced by reacting to the decrease in cellular energy in EVTs observed with preeclampsia.

We propose the correlation chart of autophagy and HIF1α for EVTs (Figure 5). In normal pregnancy, placental oxygen concentration is around 8% (physiological hypoxia).We assumed that severe hypoxia (∼2% of oxygen concentration) occurs primarily in the first trimester preeclamptic placenta [17]. In a normal pregnancy, HIF1α-activated autophagy, serving as an energy source, enhances the invasion of EVTs via PI3K under physiological hypoxia. We previously reported that autophagy in EVTs was inhibited by soluble endoglin in preeclamptic placentas as one of the causes for the pathophysiology of preeclampsia [14], and the overexpression of HIF1α was significantly higher in placental biopsies from preeclampsia than in normal placentas [32]. As severe placental hypoxia, which contributes to continued HIF1α production, accelerates soluble endoglin expression from villi, the overexpression of HIF1α may impair autophagy in EVTs by soluble endoglin, especially in the preeclamptic placenta. As a result, a decrease in cellular ATP levels in EVTs may inhibit the invasion of EVTs. Thus, severe placental hypoxia not only decreases cellular energy but also contributes to autophagy inhibition in EVTs by soluble endoglin. Therefore, we propose that HIF1α and autophagy inhibition may create a vicious cycle in the progression and manifestation of preeclampsia. On the other hand, it is still unknown which specific factor initiates autophagy inhibition, or which event induces severe hypoxia in the early stages of preeclampsia. For future studies, it is most important to identify the initiating factor for preeclampsia.

In conclusion, autophagy, which serves as an energy source for trophoblast invasion, not only maintains homeostasis, but also supports trophoblast invasion in EVTs overexpressing HIF1α. An adequate complement of ATP may supply cellular energy to EVTs with HIF1α overexpression.

## Supporting Information

Figure S1 HIF1α induction by 2% O_2_ or CoCl_2_ in the autophagy-deficient EVT cell line. a) Western blots in HTR8/SVneo cells under 31.25, 62.5, 125, 250 and 500 µM CoCl2, 2% for 24 h were shown as follows: HIF1α and α-tubulin. b) Western blots in HTR8-Atg4BC74A mutant cells, the autophagy-deficient EVT cell line, under 250 µM CoCl_2_, 2% oxygen tension, or DMSO (control) for 24 h were shown as follows: HIF1α and α-tubulin. These experiments were independently performed at least three times.
(TIF)

Figure S2 P2RX7 expression was estimated by the mean fluorescent intensity in HchEpC1b-Atg4BC74A mutant cells, the autophagy-deficient EVT cell line, or HchEpC1b-mStrawberry cells, the control cell line, treated with DMSO (control: white bars) or 250 µM CoCl_2_ (black bars) for 48 h. These experiments were independently performed at least three times. N.S.: not significant
(TIF)

Figure S3 Methylpyruvate (MP) did not recover the decreased invasiveness of HTR8-ATG4BC74A cells treated with CoCl_2_. a) Invasion assays were performed with HTR8-ATG4BC74A cells, an autophagy-deficient EVT cell line, in the presence of 250 µM CoCl_2_ with or without 10 mM methylpyruvate (MP) for 48 h. The *Y*-axis indicates the number of invading cells. These experiments were independently performed at least three times.
(TIF)

## References

[1] Genbacev O, Zhou Y, Ludlow JW, Fisher SJ (1997) Regulation of human placental development by oxygen tension. Science 277: 1669–1672. 9287221 10.1126/science.277.5332.1669

[2] Jauniaux E, Hempstock J, Teng C, Battaglia FC, Burton GJ (2005) Polyol concentrations in the fluid compartments of the human conceptus during the first trimester of pregnancy: maintenance of redox potential in a low oxygen environment. J Clin Endocrinol Metab 90: 1171–1175. 15562012 10.1210/jc.2004-1513

[3] Jauniaux E, Watson A, Burton G (2001) Evaluation of respiratory gases and acid-base gradients in human fetal fluids and uteroplacental tissue between 7 and 16 weeks' gestation. Am J Obstet Gynecol 184: 998–1003. 11303211 10.1067/mob.2001.111935

[4] Tuuli MG, Longtine MS, Nelson DM (2011) Review: Oxygen and trophoblast biology—a source of controversy. Placenta 32 Suppl 2S109–118. 21216006 10.1016/j.placenta.2010.12.013 PMC3682830

[5] Kaufmann P, Black S, Huppertz B (2003) Endovascular trophoblast invasion: implications for the pathogenesis of intrauterine growth retardation and preeclampsia. Biol Reprod 69: 1–7. 12620937 10.1095/biolreprod.102.014977

[6] Starzyk KA, Salafia CM, Pezzullo JC, Lage JM, Parkash V, et al (1997) Quantitative differences in arterial morphometry define the placental bed in preeclampsia. Hum Pathol 28: 353–358. 9042801 10.1016/s0046-8177(97)90135-0

[7] Redman CW (1997) Cytotrophoblasts: masters of disguise. Nat Med 3: 610–611. 9176484 10.1038/nm0697-610

[8] Moffett A, Loke C (2006) Immunology of placentation in eutherian mammals. Nat Rev Immunol 6: 584–594. 16868549 10.1038/nri1897

[9] Mizushima N, Ohsumi Y, Yoshimori T (2002) Autophagosome formation in mammalian cells. Cell Struct Funct 27: 421–429. 12576635 10.1247/csf.27.421

[10] Yoshimori T (2004) Autophagy: a regulated bulk degradation process inside cells. Biochem Biophys Res Commun 313: 453–458. 14684184 10.1016/j.bbrc.2003.07.023

[11] Poellinger L, Johnson RS (2004) HIF-1 and hypoxic response: the plot thickens. Curr Opin Genet Dev 14: 81–85. 15108809 10.1016/j.gde.2003.12.006

[12] Semenza GL (2007) Life with oxygen. Science 318: 62–64. 17916722 10.1126/science.1147949

[13] Caniggia I, Mostachfi H, Winter J, Gassmann M, Lye SJ, et al (2000) Hypoxia-inducible factor-1 mediates the biological effects of oxygen on human trophoblast differentiation through TGFbeta(3). J Clin Invest 105: 577–587. 10712429 10.1172/JCI8316 PMC289179

[14] Nakashima A, Yamanaka-Tatematsu M, Fujita N, Koizumi K, Shima T, et al (2013) Impaired autophagy by soluble endoglin, under physiological hypoxia in early pregnant period, is involved in poor placentation in preeclampsia. Autophagy 9: 303–316. 23321791 10.4161/auto.22927 PMC3590252

[15] Zhang H, Bosch-Marce M, Shimoda LA, Tan YS, Baek JH, et al (2008) Mitochondrial autophagy is an HIF-1-dependent adaptive metabolic response to hypoxia. J Biol Chem 283: 10892–10903. 18281291 10.1074/jbc.M800102200 PMC2447655

[16] Ietta F, Wu Y, Winter J, Xu J, Wang J, et al (2006) Dynamic HIF1A regulation during human placental development. Biol Reprod 75: 112–121. 16611863 10.1095/biolreprod.106.051557

[17] Lai Z, Kalkunte S, Sharma S (2011) A critical role of interleukin-10 in modulating hypoxia-induced preeclampsia-like disease in mice. Hypertension 57: 505–514. 21263114 10.1161/HYPERTENSIONAHA.110.163329 PMC3621110

[18] Rolfo A, Many A, Racano A, Tal R, Tagliaferro A, et al (2010) Abnormalities in oxygen sensing define early and late onset preeclampsia as distinct pathologies. PLoS One 5: e13288. 20967267 10.1371/journal.pone.0013288 PMC2953500

[19] Graham CH, Hawley TS, Hawley RG, MacDougall JR, Kerbel RS, et al (1993) Establishment and characterization of first trimester human trophoblast cells with extended lifespan. Exp Cell Res 206: 204–211. 7684692 10.1006/excr.1993.1139

[20] Omi H, Okamoto A, Nikaido T, Urashima M, Kawaguchi R, et al (2009) Establishment of an immortalized human extravillous trophoblast cell line by retroviral infection of E6/E7/hTERT and its transcriptional profile during hypoxia and reoxygenation. Int J Mol Med 23: 229–236. 19148547

[21] Fujita N, Hayashi-Nishino M, Fukumoto H, Omori H, Yamamoto A, et al (2008) An Atg4B mutant hampers the lipidation of LC3 paralogues and causes defects in autophagosome closure. Mol Biol Cell 19: 4651–4659. 18768752 10.1091/mbc.E08-03-0312 PMC2575160

[22] Tanida I, Minematsu-Ikeguchi N, Ueno T, Kominami E (2005) Lysosomal turnover, but not a cellular level, of endogenous LC3 is a marker for autophagy. Autophagy 1: 84–91. 16874052 10.4161/auto.1.2.1697

[23] Nakashima A, Ito M, Yoneda S, Shiozaki A, Hidaka T, et al (2010) Circulating and decidual Th17 cell levels in healthy pregnancy. Am J Reprod Immunol 63: 104–109. 20015328 10.1111/j.1600-0897.2009.00771.x

[24] Wang GL, Semenza GL (1993) General involvement of hypoxia-inducible factor 1 in transcriptional response to hypoxia. Proc Natl Acad Sci U S A 90: 4304–4308. 8387214 10.1073/pnas.90.9.4304 PMC46495

[25] Khakh BS, North RA (2006) P2X receptors as cell-surface ATP sensors in health and disease. Nature 442: 527–532. 16885977 10.1038/nature04886

[26] Chen G, Zhang W, Li YP, Ren JG, Xu N, et al (2013) Hypoxia-induced autophagy in endothelial cells: a double-edged sword in the progression of infantile haemangioma? Cardiovasc Res 98: 437–448. 23408345 10.1093/cvr/cvt035

[27] Choi JH, Lee HJ, Yang TH, Kim GJ (2012) Effects of hypoxia inducible factors-1alpha on autophagy and invasion of trophoblasts. Clin Exp Reprod Med 39: 73–80. 22816073 10.5653/cerm.2012.39.2.73 PMC3398120

[28] Lum JJ, Bauer DE, Kong M, Harris MH, Li C, et al (2005) Growth factor regulation of autophagy and cell survival in the absence of apoptosis. Cell 120: 237–248. 15680329 10.1016/j.cell.2004.11.046

[29] Trabanelli S, Ocadlikova D, Gulinelli S, Curti A, Salvestrini V, et al (2012) Extracellular ATP exerts opposite effects on activated and regulatory CD4+ T cells via purinergic P2 receptor activation. J Immunol 189: 1303–1310. 22753942 10.4049/jimmunol.1103800

[30] Alano CC, Garnier P, Ying W, Higashi Y, Kauppinen TM, et al (2010) NAD+ depletion is necessary and sufficient for poly(ADP-ribose) polymerase-1-mediated neuronal death. J Neurosci 30: 2967–2978. 20181594 10.1523/JNEUROSCI.5552-09.2010 PMC2864043

[31] Lu H, Burns D, Garnier P, Wei G, Zhu K, et al (2007) P2X7 receptors mediate NADH transport across the plasma membranes of astrocytes. Biochem Biophys Res Commun 362: 946–950. 17803959 10.1016/j.bbrc.2007.08.095

[32] Rajakumar A, Brandon HM, Daftary A, Ness R, Conrad KP (2004) Evidence for the functional activity of hypoxia-inducible transcription factors overexpressed in preeclamptic placentae. Placenta 25: 763–769. 15451190 10.1016/j.placenta.2004.02.011

