# Supplementary figures and images for: Autophagy Induced by HIF1α Overexpression Supports Trophoblast Invasion by Supplying Cellular Energy
Source: PLoS One. 2013 Oct 3;8(10):e76605. doi: 10.1371/journal.pone.0076605 (PMC3789701; doi:10.1371/journal.pone.0076605)

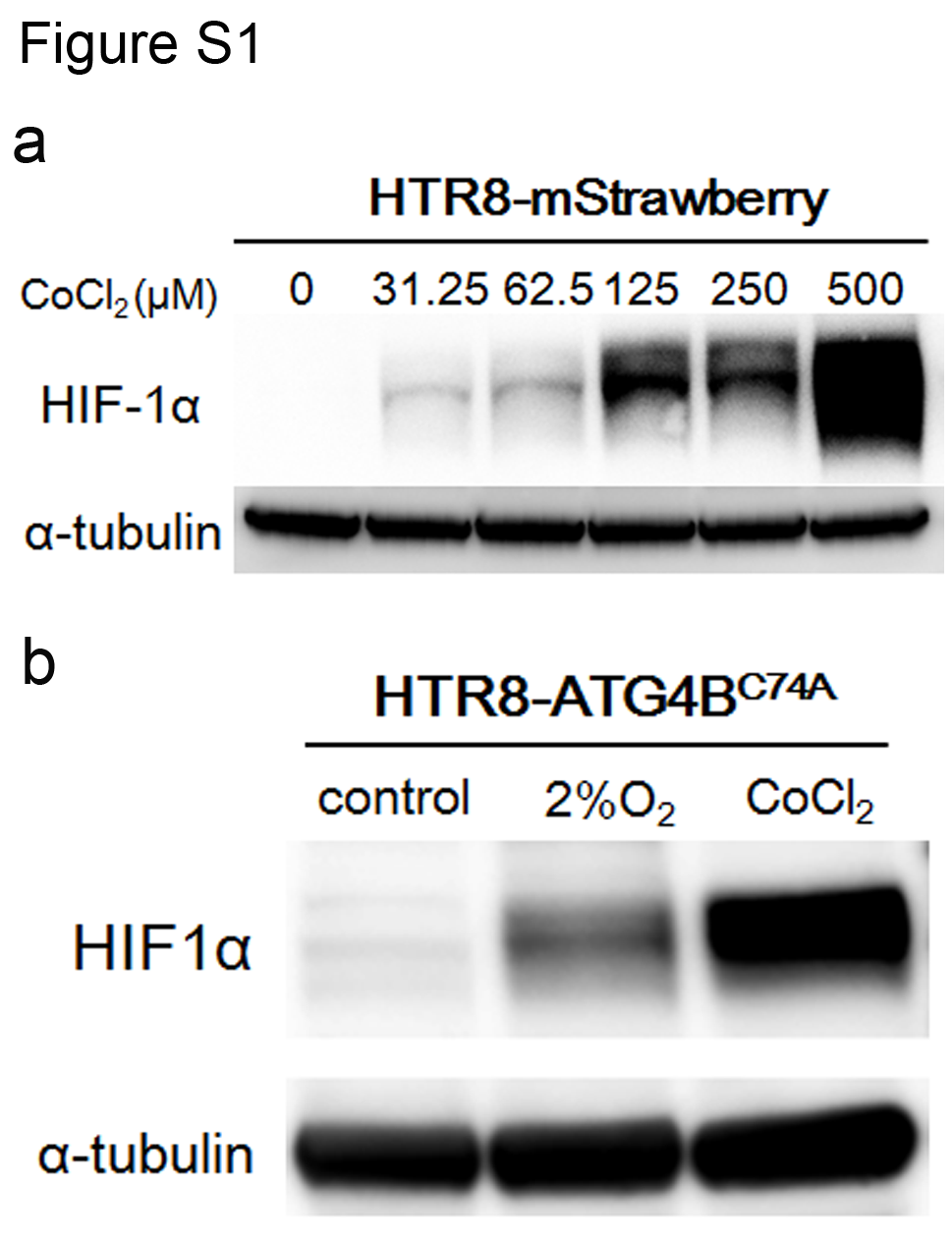

Supplement: Figure S1 — HIF1α induction by 2% O2 or CoCl2 in the autophagy-deficient EVT cell line. a) Western blots in HTR8/SVneo cells under 31.25, 62.5, 125, 250 and 500 µM CoCl2, 2% for 24 h were shown as follows: HIF1α and α-tubulin. b) Western blots in HTR8-Atg4BC74A mutant cells, the autophagy-deficient EVT cell line, under 250 µM CoCl2, 2% oxygen tension, or DMSO (control) for 24 h were shown as follows: HIF1α and α-tubulin. These experiments were independently performed at least three times. (TIF) [file pone.0076605.s001.tif]

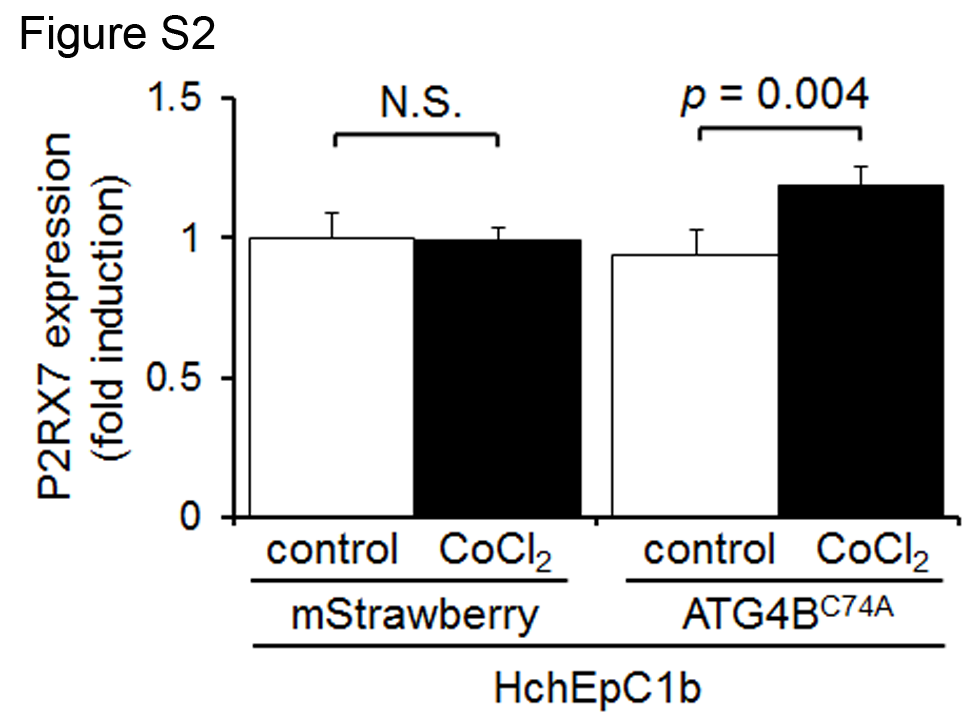

Supplement: Figure S2 — P2RX7 expression was estimated by the mean fluorescent intensity in HchEpC1b-Atg4BC74A mutant cells, the autophagy-deficient EVT cell line, or HchEpC1b-mStrawberry cells, the control cell line, treated with DMSO (control: white bars) or 250 µM CoCl2 (black bars) for 48 h. These experiments were independently performed at least three times. N.S.: not significant (TIF) [file pone.0076605.s002.tif]

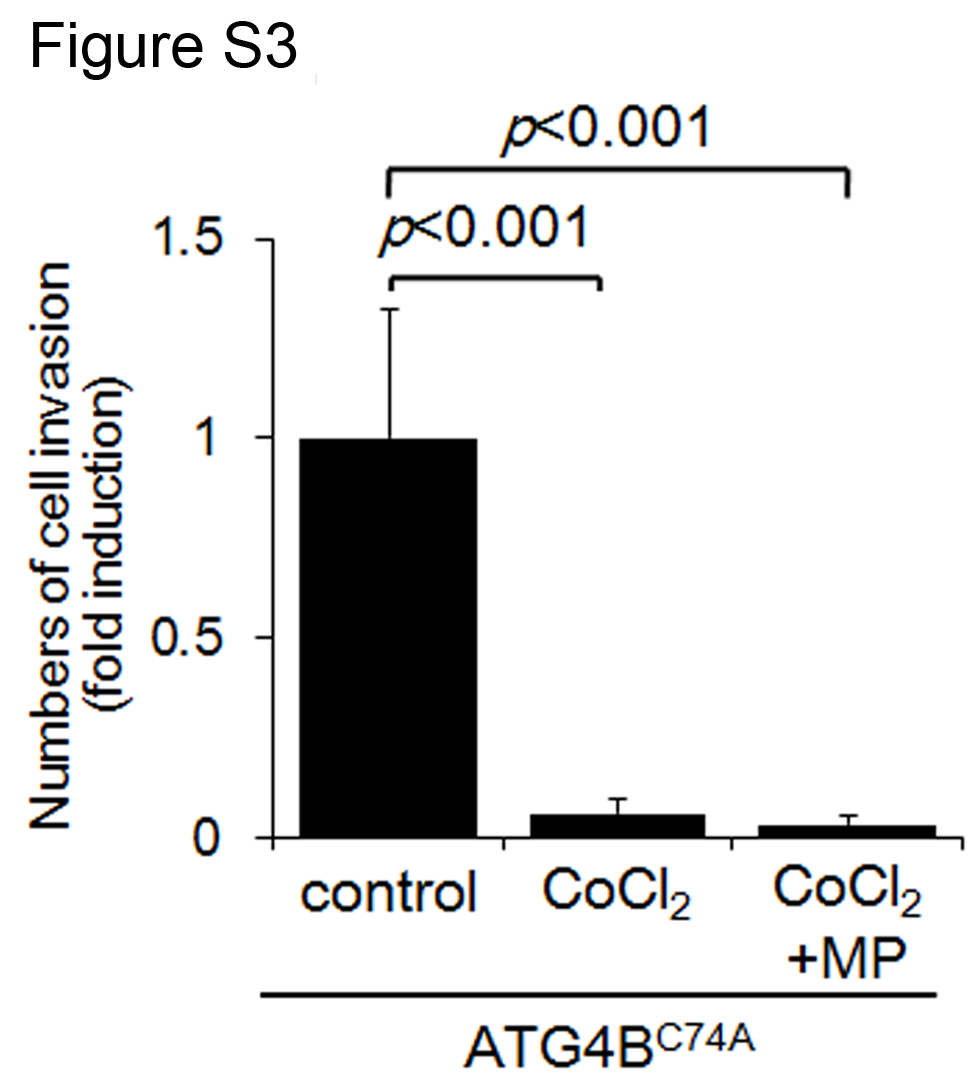

Supplement: Figure S3 — Methylpyruvate (MP) did not recover the decreased invasiveness of HTR8-ATG4BC74A cells treated with CoCl2. a) Invasion assays were performed with HTR8-ATG4BC74A cells, an autophagy-deficient EVT cell line, in the presence of 250 µM CoCl2 with or without 10 mM methylpyruvate (MP) for 48 h. The Y-axis indicates the number of invading cells. These experiments were independently performed at least three times. (TIF) [file pone.0076605.s003.tif]
